# Are the Acute Effects of THC Different in Aging Adults?

**DOI:** 10.3390/brainsci11050590

**Published:** 2021-05-01

**Authors:** Raeghan L. Mueller, Jarrod M. Ellingson, L. Cinnamon Bidwell, Angela D. Bryan, Kent E. Hutchison

**Affiliations:** 1 Department of Psychology and Neuroscience, University of Colorado Boulder, Boulder, CO 80309, USA; angela.bryan@colorado.edu (A.D.B.); kent.hutchison@cuanschutz.edu (K.E.H.); 2 Department of Psychiatry, University of Colorado Anschutz Medical Campus, Aurora, CO 80045, USA; jarrod.ellingson@cuanschutz.edu; 3 Institute of Cognitive Science, University of Colorado Boulder, Boulder, CO 80301, USA; lcb@colorado.edu

**Keywords:** cannabis, aging adults, cognition

## Abstract

In recent years of expanding legalization, older adults have reported the largest increase in cannabis use of any age group. While its use has been studied extensively in young adults, little is known about the effects of THC in older adults and whether the risks of cannabis might be different, particularly concerning intoxication and cognition. The current study investigated whether age is associated with the deleterious effects of THC on cognitive performance and other behavioral measures before and after ad libitum self-administration of three different types of cannabis flower (THC dominant, THC + CBD, and CBD dominant). Age groups consisted of young adults (ages 21–25) and older adults (ages 55–70). Controlling for pre-use scores on all measures, the THC dominant chemovar produced a greater deleterious effect in younger adults compared with older adults in tests of learning and processing speed, whereas there were no differences between old and young in the effects of the other chemovars. In addition, the young group reported greater cannabis craving than the older group after using the THC chemovar. Consistent with some reports in the preclinical literature, the findings suggest that older adults may be less sensitive to the effects of THC on cognitive and affective measures.

## 1. Introduction

Recent large-scale epidemiological research suggests that older adults increased their use of cannabis at astounding rates from 2007 to 2016 [1]. Notably, this same period also saw the first states legalize recreational cannabis. To put this unprecedented shift in perspective, 34% of the U.S. population gained legal access to recreational cannabis in less than ten years, and 68% now have legal access to medicinal cannabis. Two additional sociodemographic shifts on the horizon emphasize the need to understand how cannabis affects older adults specifically. First, a record proportion of Americans will be in older adulthood in the next 10–20 years [2]. Second, additional states will likely legalize cannabis in the coming years, thereby further increasing the number of older adults with legal access to cannabis. 

There is extensive literature examining the effects of cannabis across adolescence and young adulthood. For example, laboratory-based studies of adults have identified acute effects of cannabis use on cognitive impairment, positive mood, anxiety, and subjective reward [3,4,5,6,7,8,9,10]. Furthermore, a large meta-analysis recently found that the cognitive impairment may persist for up to 72 h after last use, but effects appear to wane thereafter [11]. Conversely, there is a notable dearth of empirical evidence on how cannabis use affects older adults and whether these effects may differ in adolescents and emerging adults. Evidence from animal models suggests that low dose delta-9-tetrahydrocannabinol (THC) may be neuroprotective in older age but harmful in younger age [12,13]. The pre-clinical research is consistent with a recent study in humans that found no difference in brain morphometry or cognitive function between older cannabis users and non-users [14]. 

There are considerable public health implications related to cannabis use in the aging population, specifically concerning their cognitive health and physical well-being. The aging population is at higher risk for neurodegeneration, cognitive decline [15], depression and anxiety [16], and other physical ailments such as chronic pain [17] and autoimmunity [18]. Aging is also associated with progressively deleterious changes in the immune system that are thought to contribute to the pathogenesis of age-related diseases, including cognitive decline [19,20,21]. Therefore, comorbidities that induce neuroinflammation and accumulating neurological damage may play a mediating role in age-related cognitive dysfunction [22]. THC is the principal psychotropic constituent of cannabis and is primarily responsible for not only its rewarding properties [23] but also for intoxication and impairment [24,25]. Given that older adults exhibit the greatest increase in cannabis use among all age groups [26,27,28], researchers must assess potential risks within this population, including acute THC intoxication, dizziness, and risk for falling. With expanding legalization and availability of cannabis for adult use, experimental observations and research studies concerning the impact of cannabis and varying cannabinoid potencies on cognitive impairment in the aging population are critical.

To examine these timely and critical research questions, the current study examined whether the acute effect of THC differed among young adults (ages 21–25) and older adults (ages 55–70). Participants provided baseline measures of blood cannabinoid levels, cognitive functioning, and subjective mood. Five days following the baseline assessment, participants were assessed during an acute experimental session where they self-administered one of three cannabis flower products in their home and in a state with legal cannabis legislation. 

During the experimental session, they were observed before, immediately after, and one hour after self-administration on measures of cannabinoid blood levels, neurocognitive functioning, and subjective measures of intoxication, anxiety, and cannabis craving. We conducted repeated measures analysis of variance to determine the effects of age (younger vs. older) and cannabis chemovar on cognitive and subjective variables of interest at post-use and 1-h post-use while controlling for pre-use scores. Based on the studies described above, we hypothesized that the young group would demonstrate more profound deleterious effects of THC on cognition, mood, and craving. 

## 2. Materials and Methods

### 2.1. Participants and Procedures

The study was approved by the University of Colorado Boulder Institutional Review Board, and written informed consent was obtained from every participant. Recreational cannabis users living in the Boulder–Denver area in Colorado were recruited through mailed flyers and screened over the phone for eligibility criteria. Participants were eligible to participate in the study if they were between 21 and 70 years of age, used cannabis flower at least four times per month for the previous six months, and self-reported using the highest concentrated cannabis flower that could be assigned in the study (i.e., 24% THC). Participants were excluded if they were daily tobacco users, heavy alcohol users (>3 drinking days per week and >5 drinks per drinking occasion) or reported having or receiving treatment for a psychotic disorder. All eligible participants were scheduled for a baseline appointment within one week of the phone screen. At their baseline visit, participants tested negative on a urine toxicology screen for recreational drug use (other than cannabis), and female participants were not pregnant or planning to become pregnant. At both the baseline and follow-up visits, participants were breathalyzed to ensure they had no measurable level of blood alcohol (i.e., a breath alcohol level of 0). A total of *n* = 159 of participants were enrolled in the study, from which young adults (ages 21 to 25, *n* = 54) and older adults (ages 55 to 70, *n* = 32) were subsampled and exclusively used in the current analysis (*n* = 86). Participants were subsampled based on their age to determine whether the acute effects of smoking cannabis flower comprising three varying THC-to-CBD ratios differ between young and older adults.

#### 2.1.1. Baseline Session

Participants completed a series of questionnaires that recorded demographic information, past and current substance use, medical history, health status, cognition, and current mood, and a trained phlebotomist collected intravenous blood. At the end of the appointment, participants were randomly assigned to the letter (i.e., “A”, “B” or “C”) using a random assignment table generated by the study staff. Each letter corresponded to a specific flower product tested from an International Organization of Standards (ISO) 17025 accredited laboratory at either 24% THC (<1% CBD), 23% CBD (<1% THC), or a product with a similar concentration of THC (9%) and CBD (10%). Participants were provided directions to a local, study-partnered dispensary (The Farm; available online: http://thefarmco.com (accessed on 15 January 2021)) and instructed to purchase enough product to use for the following five days. Participants were asked to exclusively use this study product for the following 5 days ad libitum to familiarize themselves with the product before their follow-up appointment.

#### 2.1.2. Follow-Up Session

An assessment of the acute effects of cannabis was performed in a mobile pharmacology lab that was driven by two researchers to the participant’s home. Participants were asked to abstain from using any form of cannabis on the day of the appointment. During the first mobile laboratory assessment (pre-use), participants completed cognitive measures and subjective questionnaires, and provided a blood sample. Following the pre-use assessment, participants returned to their home to use their assigned cannabis chemovar ad libitum using their preferred method of administering cannabis flower via inhalation. Participants were provided a scale and asked to weight and record the weight of their flower product (in grams) before and after administration (see Table 1). Upon returning to the mobile laboratory, participants completed the same cognitive tasks and subjective questionnaires, and provided a second blood sample (acute post-use). Participants remained in the mobile laboratory to complete all measures a third and final time exactly 1 h later (1 h post-use). 

### 2.2. Measures

#### 2.2.1. Demographics and Substance Use

Participants completed a questionnaire at their baseline visit that collected information on their age, sex, and race, and answered a question regarding how old they were when they first started using cannabis regularly (i.e., at least once per week). Cannabis use disorder (CUD) symptoms were assessed using the 11-item CUDS, a measure of cannabis dependence severity [26]. Participants also completed Timeline Followback (TLFB), a retrospective recall measure of daily cannabis, alcohol, and other substance use [28]. The TLFB was used to evaluate and quantify substance use for the 30-days before the baseline appointment and for the 5-days prior to the follow-up.

#### 2.2.2. Assessment of Blood Cannabinoids Levels

Whole blood (4 mL) was collected from each participant through venipuncture of a peripheral arm vein using standard, sterile phlebotomy techniques at baseline and at each timepoint at follow-up by a certified phlebotomist. Blood was stored at 4 °C for the duration of the follow-up appointment in a temperature-controlled cooler and returned to the on-campus research facility at the end of the appointment. Upon return to the on-campus facility, blood was centrifuged (1000× *g*, 10 min) and plasma aliquoted into amber glass vials and stored at −80 °C. Plasma samples were sent to the iC42 Clinical Research and Development (Department of Anesthesiology) on the Anschutz Medical Campus at the University of Colorado Denver. We quantified THC using validated high-performance liquid chromatography/mass-spectroscopy (HPLC-MS/MS) (API5500) [28].

#### 2.2.3. Cognitive Performance

Cognitive performance and functioning were measured before and after cannabis self-administration during the follow-up experimental session. Participants completed four cognitive tasks administered through the NIH Toolbox iPad Application [29]. The four tasks included the picture sequence memory (PSM) test, pattern comparison processing speed (PCPS) test, the dimensional change card sort (DCCS) test, and the flanker inhibitory control and attention (FICA) test. Each task assessed episodic memory, processing speed, executive functioning, and attention, respectively, and scores were age- and sex-normed. 

#### 2.2.4. Subjective Intoxication

To assess subjective intoxication before and after cannabis self-administration, participants completed a 1-item measure that stated, “I feel high (as in “drug high”) right now”, to which they rated on an 11-point scale from 0 (not at all) to 10 (strongest feeling possible).

#### 2.2.5. Subjective Craving

A 4-item Marijuana Craving Questionnaire (MCQ) was used to assess participants’ current craving for cannabis at baseline (α = 0.89), pre-use (α = 0.92), post-use (α = 0.87), and 1-h post-use (α = 0.89). Participants rated on a scale from 0 (not at all) to 10 (strongest feeling possible) items such as “I have a desire to use marijuana right now” and “I crave marijuana right now”. Question items were averaged to create one MCQ score. 

#### 2.2.6. Subjective Anxiety

Participants completed a modified version of the Profile of Mood States (POMS) [30], a self-report questionnaire consisting of 21 adjectives used to describe momentary mood states. The POMS was used to assess subjective anxiety and tension at each of the three timepoints at follow-up. Participants indicated how they felt at the moment concerning each adjective on a 5-point scale from 0 (Not at all) to 4 (Extremely). Scores from six individual POMS items were averaged to create one subscale that assessed subjective anxiety and tension (e.g., paranoid, anxious, tense, nervous, unable to relax, and shaky). 

#### 2.2.7. Subjective Dizziness

A 1-item measure was used to assess whether participants felt dizzy immediately after and 1-h after cannabis self-administration. Participants rated on a 5-point scale from 0 (not at all) to 4 (extremely) on the question “Do you feel dizzy?” from the effects of cannabis.

### 2.3. Statistical Analysis

A total of *n* = 159 of participants were enrolled and classified into one of two age groups based on whether they were between the ages of 21 and 25 (younger age group, *n* = 54) or between the ages of 55 and 70 (older age group, *n* = 32). Only those participants who fell within either age group were included in the present analysis (*n* = 86). Independent samples t-tests and chi-square tests were used to examine age group differences on continuous and categorical demographic and substance use variables, respectively. See Table 1 for additional sample details and group differences. 

Three-way repeated-measures analysis of variance (RMANOVA) models were used to determine the effects of age (younger vs. older), chemovar (THC vs. CBD vs. THC + CBD), and time on cognitive and subjective variables of interest at acute post-use and 1-h post-use, while controlling for their respective pre-use scores at follow-up. One three-way RMANOVA was run for plasma THC (ng/mL) levels, subjective intoxication, cannabis craving, and subjective anxiety, and four RMANOVAs were conducted on the four separate cognitive performance tasks. Lastly, an analysis of covariance (ANCOVA) was used to determine the effects of age and chemovar on subjective dizziness at 1-h post-use, controlling for acute post-use dizziness. 

Significant main effects of age or chemovar were followed up by examining the simple effects of age within each level of chemovar, and the simple effects of chemovar within each level of age, respectively. A significant age-by-chemovar interaction effect was followed up by examining pairwise comparisons for all possible simple effects, and *p*-values Bonferroni adjusted. Statistical analyses were carried out in the software package IBM Statistical Package for the Social Sciences (SPSS version 27.0, SPSS Inc., Chicago, IL, USA). Graphs were created using GraphPad Prism 8 (GraphPad, San Diego, CA, USA). 

## 3. Results

### 3.1. Sample Characteristics

Eighty-six participants were included in the present analysis and were either between ages of 21 and 25 (*n* = 54, mean (*M*) = 22.48 years, standard deviation (*SD*) = 1.37) or between the ages of 55 and 70 (*n* = 32, *M* = 63.13, *SD* = 4.66). There were no significant differences in gender or race among the two age groups. The age onset of regular cannabis use (i.e., once per week) was significantly different between age groups (*t*_(84)_ = −3.397, *p* = 0.002), where the older group (*M* = 27.22, *SD* = 16.25) started using cannabis regularly at a later age compared with the younger group (*M* = 17.41, *SD* = 2.25). Also, the younger age group (*M* = 3.59, *SD* = 2.62) had a significantly higher mean CUD score than the older age group (*M* = 1.53, *SD* = 2.38) (*t*_(84)_ = 3.648, *p* = 0.0001).

Using the data collected from the 30-day TLFB, there were no significant age group differences regarding participants’ total number of cannabis use days in the past 30-days, which included combining any day they used cannabis flower, edibles, or concentrated cannabis. However, the older age group reported more edible use days than the younger age group (*t*_(84)_ = 3.579, *p* = 0.001). No group differences emerged for the total number of flower or concentrate use days in the past 30-days. Everyone in the younger age group reported at least two cannabis use days in the past 30-days, 98% of which reported at least one flower use day, 33% at least one edible use day, and 62% reported at least one concentrate use day. Everyone in the older age group reported at least one or more cannabis use days in the past 30-days, 84% of which reported at least one flower use day, 43% at least one edible use day, and 34% reported one or more concentrate use days. See Table 1 for additional sample details and pre-use cognitive performance and subjective response scores. 

### 3.2. Effects of Age on Blood Plasma THC Levels

**Figure 1:**
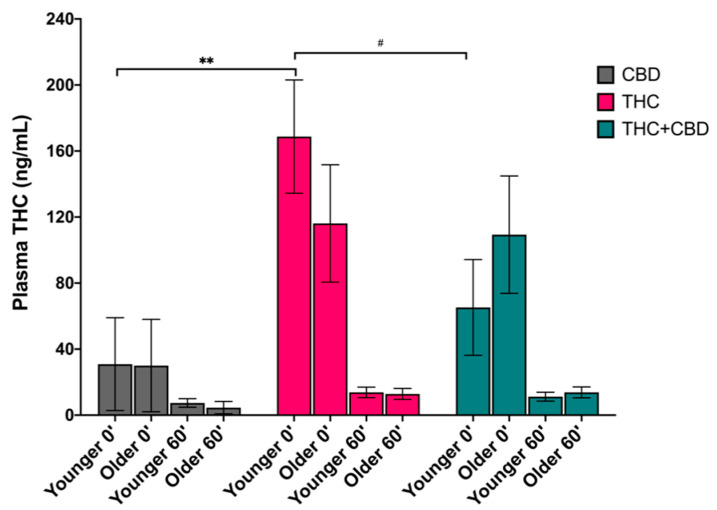
Blood plasma THC levels (ng/mL) immediately following cannabis self-administration (post-use, 0′) and 1-h later (60′) by age group (younger vs. older) and by chemovar condition (CBD, gray; THC, pink; THC + CBD, blue), controlling for pre-use blood levels. There was a simple effect of chemovar within the younger age group, such that post-use (0′) THC levels were significantly different between the THC and CBD conditions within the younger age group (*p* = 0.009), and a trending significant difference between the THC and THC + CBD conditions (^#^ *p* = 0.074). The data represent the adjusted mean + standard error of the adjusted mean (SEM) for each age group within each chemovar condition. ^#^ *p* < 0.1, ** *p* < 0.01.

A three-way repeated-measure analysis of variance (RMANOVA) was conducted to determine the effects of age, chemovar, and time on plasma THC levels (ng/mL) at acute post-use and 1-h post-use, controlling for pre-use THC levels. There was a significant main effect of chemovar on plasma THC levels (*F*_(2,63)_ = 4.927, *p* = 0.01, partial η^2^ = 0.135), such that averaging across age and time, individuals in the THC condition (*M* = 77.88, *SEM* = 13.36) had higher plasma THC levels than those in the CBD condition (*M* = 18.23, *SEM* = 13.25), a significant difference of 59.66 (*p* = 0.008). There was a simple chemovar effect within the younger age group, such that post-use THC blood levels were significantly higher in young adults using the THC versus CBD chemovar (mean difference of 137.85, *p* = 0.009). Additionally, a marginally significant difference between the THC and THC + CBD chemovar conditions within the young adult group emerged (mean difference of 103.49, *p* = 0.074). See Figure 1. 

### 3.3. Effects of Age and Chemovar on Cognitive Performance

**Figure 2:**
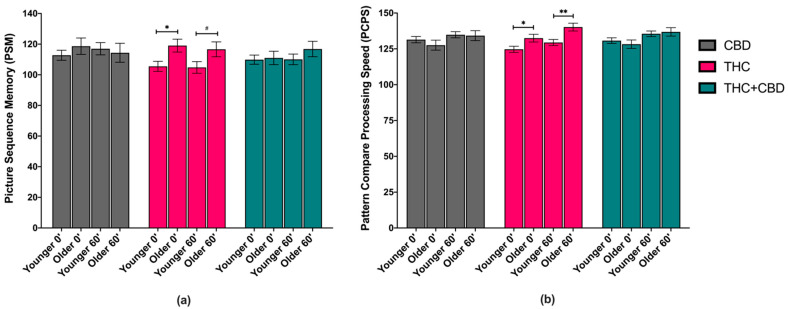
Cognitive performance scores immediately following cannabis self-administration (post-use, 0′) and 1-h later (60′) by age group (younger vs. older) and by chemovar (CBD, gray; THC, pink; THC + CBD, blue), while controlling for pre-use scores. (**a**) Picture sequence memory (PSM) task. There was a simple effect of age on post-use (0′) PSM scores within the THC condition, such that the younger age group (Younger 0′) performed worse on this task compared with the older age group (Older 0′) (*p* = 0.014), and a trending significant difference at 1h post-use (60′) (*p* = 0.061). (**b**) Pattern comparison processing speed (PCPS) task. There was a simple effect of age on post-use (0′) PCPS scores within the THC condition, such that the younger age group (Younger 0′) performed worse on this task compared with the older age group (Older 0′) (*p* = 0.029). Similarly, there was a significant simple effect of age group on 1-h post-use PCPS scores (60′) within the THC condition, where again, the younger age group performed worse on this task than the older age group (*p* = 0.003). The data represent the adjusted mean + standard error of the adjusted mean (SEM) for each age group within each chemovar condition. ^#^ *p* < 0.1, * *p* < 0.05, ** *p* < 0.01.

RMANOVA revealed a significant main effect of age on PSM scores (*F*_(1,69)_ = 4.541, *p* = 0.037, partial η^2^ = 0.062). Averaging across chemovar condition and time, the younger age group (*M* = 109.96, *SEM* = 1.62) performed worse on this task than the older age group (*M* = 116.08, *SEM* = 2.37). There was a simple age effect within the THC chemovar condition, where post-use PSM scores were significantly lower in the younger versus older age group, a significant difference of 13.53 (*p* = 0.014), and a trending simple age effect at 1 h post-use (mean difference of 11.79, *p* = 0.061). No simple age effects were observed within the THC + CBD or CBD chemovar conditions at either timepoint. See Figure 2a. 

RMANOVA revealed a significant age by chemovar interaction on PCPS scores (*F*_(1,69)_ = 3.512, *p* = 0.035, partial η^2^ = 0.092). Pairwise comparisons revealed a simple age effect within the THC chemovar condition on post-use and 1h post-use PCPS scores, such that the younger age group performed worse on this task immediately after (*F*_(1,69)_ = 5.004, *p* = 0.029, partial η^2^ = 0.068) and 1-h after self-administering the THC chemovar (*F*_(1,69)_ = 9.614, *p* = 0.003, partial η^2^ = 0.122) than the older age group. See Figure 2b. 

Lastly, there were no significant main effects or interaction effects observed on the dimensional change card sort (DCCS) test or flanker inhibitory control and attention (FICA) test, which assessed executive function and attention, respectively. 

### 3.4. Effects of Age and Chemovar on Subjective Responses

#### 3.4.1. Intoxication

There was a significant main effect of chemovar on subjective intoxication (*F*_(1,74)_ = 14.326, *p* < 0.0001, partial η^2^ = 0.279). Averaging across age group and time, individuals using the THC chemovar reported feeling “higher” (*M* = 5.76, *SEM* = 0.38) than those using the CBD chemovar (*M* = 2.98, *SEM* = 0.38), a significant mean difference of 2.78 (*p* = 0.0001). Further, those who used the THC + CBD chemovar (*M* = 5.02, *SEM* = 0.35) reported feeling “higher” than those in the CBD condition, a significant mean difference of 2.04 (*p* = 0.001). No significant age main effect or age-by-chemovar interaction emerged.

#### 3.4.2. Craving

**Figure 3:**
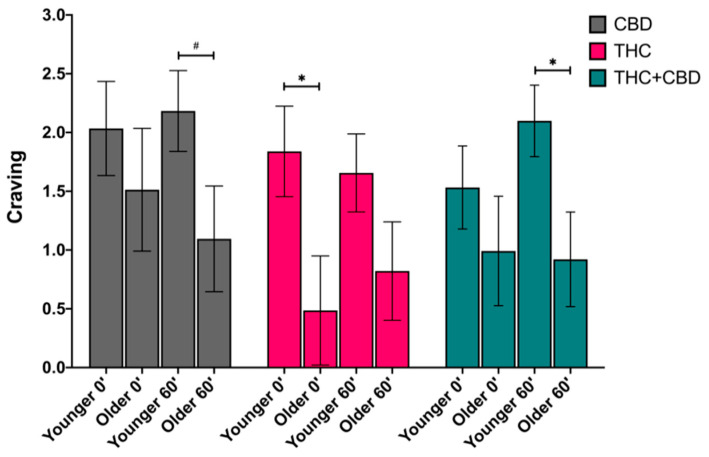
Subjective cannabis craving immediately following self-administration (post-use, 0′) and 1-h later (60′) by age group (younger vs. older) and by chemovar condition (CBD, gray; THC, pink; THC + CBD, blue), controlling for pre-use craving. There was a simple effect of age on post-use (0′) craving within the THC condition, such that the younger age group (Younger 0′) craved cannabis more than the older age group (Older 0′) (*p* = 0.03). Similarly, the younger age group (Younger 60′) craved cannabis more than the older group (Older 60′) 1-h after self-administering the THC + CBD product (*p* = 0.022). Lastly, there was a trending simple effect of age on 1h post-use craving within the CBD condition, where again, the younger group reported greater craving (*p* = 0.065). The data represent the adjusted mean + standard error of the adjusted mean (SEM) for each age group within each chemovar condition. ^#^ *p* < 0.1, * *p* < 0.05.

There was a significant main effect of age on cannabis craving (*F*_(1,74)_ = 8.59, *p* = 0.004, partial η^2^ = 0.104). Averaging across chemovar and time, the younger age group (*M* = 1.89, *SEM* = 0.19) craved cannabis more than the older age groups (*M* = 0.97, *SEM* = 0.25). The difference in cannabis craving between the younger and older age group was significant within the THC condition at post-use (mean difference of 1.38, *p* = 0.03), after 1-h post-use within the THC + CBD condition (mean difference of 1.18, *p* = 0.02), and marginally significantly different after 1h within the CBD condition (mean difference of 0.52, *p* = 0.065). See Figure 3.

#### 3.4.3. Anxiety

**Figure 4:**
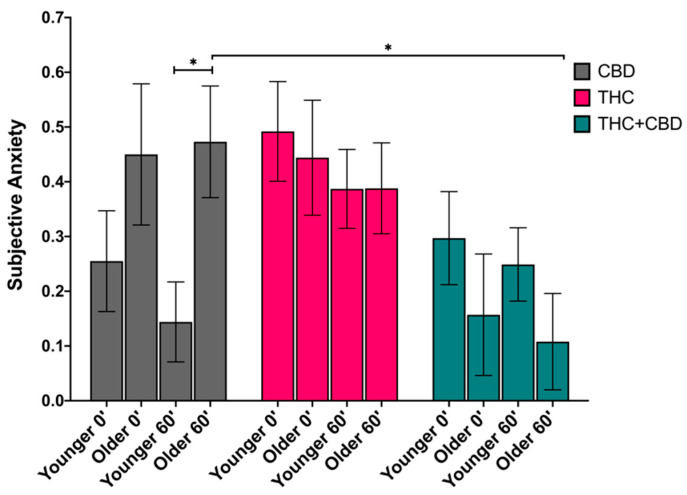
Subjective anxiety immediately following self-administration (post-use, 0′) and 1h later (60′) by age group (younger vs. older) and by chemovar condition (CBD, gray; THC, pink; THC + CBD, blue), controlling for pre-use craving. There was a simple effect of age on 1h post-use (60′) anxiety within the CBD condition, such that the older age group (Older 60′) felt more anxious than the younger age group (Younger 60′) (*p* = 0.01). There was a simple effect of chemovar within the older age group at 1-h post-use (Older 60′), revealing that older adults who used the CBD chemovar felt more anxious than the older adults who smoked the THC + CBD chemovar (*p* = 0.025). The data represent the adjusted mean + standard error of the adjusted mean (SEM) for each age group within each chemovar condition. * *p* < 0.05.

There was a significant main effect of chemovar on subjective anxiety (*F*_(2,76)_ = 4.023, *p* = 0.022, partial η^2^ = 0.096), such that averaging across age and time, individuals in the THC condition (*M* = 0.43, *SEM* = 0.06) felt more anxious than those in the THC + CBD (*M* = 0.2, *SEM* = 0.06), a significant difference of 0.23 (*p* = 0.018). There was also a significant age-by-chemovar interaction on subjective anxiety (*F*_(2,76)_ = 3.512, *p* = 0.047, partial η^2^ = 0.077). Pairwise comparisons revealed a significant simple age effect on 1-h post-use subjective anxiety within the CBD condition (*F*_(1,76)_ = 6.974, *p* = 0.01, partial η^2^ = 0.084), such that the older age group (*M* = 0.47, *SEM* = 0.1) felt more anxious than the younger age group (*M* = 0.14, *SEM* = 0.07) 1-h after using the CBD product. There was also a significant simple chemovar effect within the older age group at 1-h post-use (*F*_(2,76)_ = 4.358, *p* = 0.016, partial η^2^ = 0.1), revealing that older adults who used the CBD chemovar (*M* = 0.47, *SEM* = 0.1) felt more anxious than older adults who smoked the THC + CBD chemovar (*M* = 0.11, *SEM* = 0.09), a significant mean difference of 0.36 (*p* = 0.025). See Figure 4.

#### 3.4.4. Dizziness

Controlling for pre-use dizziness, ANCOVA revealed no significant effect of age on dizziness or an effect of chemovar. Further, there was not a significant age-by-chemovar interaction on post-use or 1-h post-use dizziness. 

## 4. Discussion

The current study investigated the effects of age on cognitive performance after ad libitum self-administration of three widely available cannabis flower products. Age groups consisted of young adults (ages 21–25) and older adults (ages 55–70), and the three chemovars comprised different concentrations of THC and CBD. While controlling for cognitive performance scores before self-administration, our findings suggest that THC has a more deleterious impact on young adults’ cognitive functioning. Using the picture sequence memory (PSM) and pattern comparison (PCPS) task, THC demonstrated a greater adverse impact on learning and processing speed in younger adults than in older adults. However, we did not find any age effects on the dimensional change card sort (DCCS) test or the flanker inhibitory control and attention (FICA) test, which assessed attention and executive functioning. Our observations suggest that THC impacts learning, memory, and processing speed in younger individuals more so than in older adults but has a less deleterious influence on processes involving attention and executive function. 

Another similarly designed study of young adults self-administering cannabis ad libitum and at similar THC potencies to the current study (12.5 THC) found acute effects on cognition and increased arousal and positive mood [31]. They found acute cognitive performance was hindered on a verbal learning task after using the higher THC product, but that same group improved on an associative learning task (Digit Symbol Substitution Test (DSST)) 48 h later [31]. Although this study utilized different cognitive tasks and did not have an older comparison group, it revealed ad libitum administration of THC flower acutely impacts verbal learning and cognitive function in young adults. 

In addition to assessing the impact of THC on cognitive performance, we investigated the effect of age on subjective measures of cannabis craving and anxiety. While controlling for pre-use craving and anxiety, we found that young adults craved cannabis more than older adults after using the THC product. Our observation on cannabis craving suggests young adults may be more susceptible than older adults to drug-seeking when using higher-concentrated THC products. One possible explanation for reduced cannabis craving in older adults could be due to differences in cannabis use disorder symptoms as individuals in the younger age group had higher symptoms of CUD than those in the older age group on average (see Table 1). Interestingly, there were no differences between the older and younger participants with respect to the effects of the high THC chemovar on anxiety and dizziness. 

It is important to note that the younger and older groups did not differ on blood concentrations of THC after using the THC dominant chemovar, nor did the age groups report different levels of subjective intoxication. These findings suggest that the differences in cognitive performance between the two groups were not merely a result of different THC blood levels or subjective high. Prior research suggests that older adults have an impaired and slower metabolism and an increased risk of experiencing THC-related effects [32,33]. Based on that observation alone, the older age group might be expected to demonstrate greater effects of THC. However, younger individuals appeared to be more sensitive to the effects of THC on cognitive and behavioral measures, which is consistent with decades of research suggesting that adolescents and emerging adults may be more sensitive to the effects of THC during critical periods of brain development [34]. 

Interestingly, this study’s younger age group had more experience with using higher-concentrated THC products and reported using cannabis regularly at a younger age. Based on prior experience, one would expect the younger age group to have developed a greater tolerance to THC and be less impacted by the psychoactive effects of THC. Although the literature is limited in aging adults, the findings are also consistent, with some preclinical research suggesting that young individuals might be more susceptible to the deleterious effects of THC as compared with older individuals [13]. In addition, it has been well documented that there are age-dependent changes in the endocannabinoid system (ECS) and that both cannabinoid receptors (CBR) and endocannabinoid (eCB) expression levels diminish with age [35,36,37,38]. Thus, our age group differences may be driven by differential expression of CBRs, specifically cannabinoid receptor type 1 (CB1) and eCB activity in brain regions governing executive function and reward. Overall, our findings would suggest THC has an adverse impact on aspects of cognition in younger adults and that young adults are more susceptible to phenotypes of THC dependence. 

## 5. Conclusions

The current study was exploratory and had several limitations that should be considered. The participant data utilized in this study were subsampled from a larger study, and age groups obtained from the large participant sample were small. Additionally, dosing and administration methods were not controlled because federal law prohibits the experimental administration of cannabis legally available in state-regulated markets. 

However, given the public health implications of this study, focused research is clearly needed on the effects of cannabis products in the aging population, as they are at higher risk for neurodegeneration and cognitive decline. Notably, recent epidemiological research indicates the greatest increase in cannabis use rates in the older adult population. Therefore, with expanding legalization and availability of cannabis, experimental observations concerning the impact of cannabis on cognitive impairment in the aging population are critical. In particular, studies need to be conducted in adults aged 65 and older and should include measures that may reflect other potential risks of cannabis in this population (e.g., dizziness, vertigo, balance, motor function, and driving). Importantly, given the well-documented age-dependent changes in the ECS, future studies should examine whether age-related changes in the ECS mediate the effect of THC and other cannabinoids.

## Figures and Tables

**Table 1:** Demographics, sample characteristics, and pre-use cognitive performance and subjective response scores among age groups and chemovar conditions.

Characteristic [Mean (SD)]	Overall (*n* = 86)		CBD (*n* = 26)		THC (*n* = 30)		THC + CBD (*n* = 30)	
	Younger (*n* = 54)	Older (*n* = 32)	Younger (*n* = 17)	Older (*n* = 9)	Younger (*n* = 18)	Older (*n* = 12)	Younger (*n* = 19)	Older (*n* = 11)
**Demographics**								
Age	22.45 (1.37)	63.13 (4.66) ***	22.94 (1.39)	65 (4.66)	22.11 (1.13)	61.33 (5.03)	22.42 (1.5)	63.55 (3.85)
Gender (% male)	66.7%	59.4%	58.8%	66.7%	72.8%	58.3%	68.4%	54.5%
Race (% white)	79.6%	93.8%	82.4%	100%	77.8%	91.7%	78.9%	90.9%
**Substance Use**								
Age of onset	17.41 (2.25)	27.22 (16.25) **	17.65 (2.06)	24.33 (14.83)	16.33 (1.94)	27 (16.64)	18.21 (2.39)	29.82 (17.96)
CUD Score	3.59 (2.62)	1.53 (2.38) ***	4.18 (2.69)	1 (1.11)	3.89 (2.52)	1.25 (0.62)	2.79 (2.57)	2.27 (3.9)
Cannabis use days ^a^	23.39 (6.97)	22.28 (8.14)	24.65 (6.16)	21.67 (10.52)	23.06 (7.84)	21.17 (8.93)	22.58 (7)	24 (4.89)
Flower use days ^a^	18.44 (9.94)	18.09 (11.13)	18.06 (10.46)	20.89 (10.16)	18.61 (9.57)	12.58 (12.62)	18.63 (10.33)	21.82 (8.21)
Edible use days ^a^	0.74 (1.5)	5.91 (10.47) **	0.71 (0.92)	4.22 (9.65)	1 (2.09)	7.25 (10.75)	0.53 (1.31)	5.82 (11.53)
Concentrate use days ^a^	7.56 (9.81)	4.50 (9.02)	10.29 (10.65)	1.11 (2.61)	7.28 (9.69)	7.25 (11.27)	5.37 (9.01)	4.55 (9.35)
**Follow-up Pre-Use**								
PSM ^b^	109.04 (20.67)	112.96 (16.45)	110.31 (19.37)	104.67 (19.09)	108.19 (17.39)	119.4 (13.67)	108.68 (24.88)	111.22 (16.35)
PCPS ^b^	125.67 (17.44)	115.2 (13.59)	130.5 (13.67)	119.5 (9.64)	125.19 (15.96)	116.9 (15.11)	122 (21.02)	110.44 (14.01)
DCCS ^b^	108.33 (10.68)	107.68 (12.95)	108 (10.63)	105.33 (13.95)	105.75 (9.61)	117.3 (9)	110.79 (11.54)	98.56 (8.77)
FICA ^b^	98.78 (15.96)	99.76 (10.79)	102.69 (18.96)	106.17 (8.63)	96.88 (17.86)	96.6 (11.77)	97.11 (11.09)	99 (10.13)
Craving ^c^	2.34 (2.43)	1.4 (2.02)	3.28 (2.77)	0.69 (1.08)	1.93 (2.02)	2.1 (1.89)	1.92 (2.35)	1.34 (2.6)
Anxiety ^d^	0.37 (0.6)	0.31 (0.3)	0.64 (0.93)	0.42 (0.43)	0.31 (0.32)	0.35 (0.23)	0.17 (0.2)	0.18 (0.24)
THC (ng/mL)	6.14 (9.65)	4.58 (8.79)	5.42 (6)	2.68 (5.49)	7.57 (14.31)	5.89 (12)	5.46 (2.93)	4.69 (7.17)
Grams used	0.31 (0.33)	0.23 (0.27)	0.39 (0.38)	0.11 (0.06)	0.23 (0.29)	0.32 (0.33)	0.31 (0.28)	0.23 (0.28)

Note: ^a^ Data from baseline 30-day Timeline Followback. ^b^ Cognitive tasks from the NIH Toolbox Application. PSM = Picture sequence memory test; PCPS = pattern comparison processing speed test; DCCS = dimensional change card sort test; FICA = flanker inhibitory control and ^c^ Marijuana Craving Questionnaire (MCQ) composite score. ^d^ Subjective anxiety from the Profile of Mood States (POMS) tension subscale. Significant overall age group differences from independent samples *t*-tests indicated as follows: ** *p* < 0.01, *** *p* < 0.001.

## Data Availability

The data that support the findings of this study are available from the corresponding author upon request.
